# Evidence brief on facilitators, barriers and hesitancy of COVID-19 booster doses in Canada

**DOI:** 10.14745/ccdr.v50i10a02

**Published:** 2024-10-03

**Authors:** Kaitlin M Young, Tricia Corrin, Kusala Pussegoda, Austyn Baumeister, Lisa A Waddell

**Affiliations:** 1 National Microbiology Laboratory, Public Health Agency of Canada, Guelph ON

**Keywords:** booster, COVID-19, vaccine acceptance, vaccine hesitancy

## Abstract

**Background:**

Understanding the facilitators, barriers and hesitancy to accepting COVID-19 booster doses is important for encouraging recommended vaccination. This evidence brief summarizes literature on the intention to accept or reject COVID-19 vaccine booster doses and the factors associated with intention/uptake among individuals in Canada.

**Methods:**

A database of COVID-19 literature established at the Public Health Agency of Canada was searched for articles referencing vaccination and knowledge, attitudes and behaviours towards COVID-19 boosters. A grey literature search of Canadian governmental and academic institutions was also conducted. Primary research conducted in Canada (n=21) and relevant systematic reviews of the global literature (n=8) were included in this evidence brief.

**Results:**

Intentions to get a booster dose in the general population have decreased between 2021–2023, with intentions varying across subpopulations. In Canada and within the global systematic reviews, facilitators, barriers and hesitancy were similar. Older age was the most common factor positively associated with intention/uptake of a booster, and the most common motivators were government/healthcare provider recommendations and helping to protect others. The main reasons for hesitancy were concerns about vaccine side effects and a lack of belief in the vaccine’s efficacy.

**Conclusion:**

Intentions to get a booster dose have decreased in Canada. Understanding the reasons for vaccine hesitancy and motivators for obtaining a booster can help guide future public health COVID-19 booster vaccination programs.

## Introduction

Canada has one of the highest vaccination rates for COVID-19 in the world. As of February 2024, more than 81% of the total population had received at least one dose and more than 16% of Canadians had received the most recent XBB.1.5 vaccine, which was released in October 2023 ((1)). The XBB.1.5 COVID-19 vaccine is the current version as of March 2024 and is recommended for both the primary series and as a booster (additional) dose ((2)).

Understanding the facilitators, barriers and hesitancy to accept or refuse COVID-19 booster doses among those who have already accepted their primary series is important for encouraging recommended vaccination in the face of waning immunity and more transmissible variants. This evidence brief summarizes literature, available up to January 31, 2024, on the intention and associated factors to accept or reject additional booster doses of COVID-19 vaccine among individuals in Canada. This information is also contrasted with global systematic reviews on the topic. This brief aims to identify whether there are any context-specific roots of vaccine hesitancy in Canada to guide tailored strategies and future public health vaccination campaigns.

## Methods

A continuous scan of the COVID-19 literature (published and pre-published) by the Public Health Agency of Canada has been underway since January 2020 ((3)). Standardized searches to retrieve COVID-19 literature are conducted in PubMed, Scopus and EuropePMC. The results are maintained in an Endnote™ database and are also accessible in Microsoft Excel®. To develop this brief, targeted keyword searching was conducted within these repositories to identify 1) primary research in Canada and 2) global evidence syntheses (i.e., systematic reviews, scoping reviews, rapid reviews summarizing evidence across multiple countries) on vaccination and knowledge attitudes and behaviours towards COVID-19 boosters. Search terms included: (“vaccin*” OR “immuni*”) AND (“third dose*” OR “booster” OR “fourth dose*” OR “fifth dose* OR “additional dose*”). Potentially relevant citations were screened for relevance to the evidence brief question and tagged by country of conduct to identify the Canadian research and global evidence syntheses. Each reference was examined to confirm its relevance and data was extracted by a single reviewer into Table S1 and Table S2 (see **Appendix** for details on the Supplementary Information) using an a priori developed structured format. Data extraction was verified by a senior reviewer. Research that reported only on vaccination in general or reported analysis such that booster results could not be teased apart from primary series results, were excluded. Narrative reviews and other secondary research were excluded. This evidence brief contains research published up to January 31, 2024.

A grey literature search was conducted to complement the bibliographic database search. The grey literature search focused on targeted Canadian governmental and academic institutions (Grey Literature Search S3). The grey literature search was completed on February 1, 2024.

## Results

Twenty-one Canadian studies evaluating the attitudes and acceptance of COVID-19 vaccine booster doses between August 2021 and October 2023 were identified and included in this evidence brief (Table S1). Of these, ten were published articles and 11 were reports that had not completed a journal’s peer-review process. Many of the studies were observational designs, including longitudinal surveys (n=7), cross-sectional studies (n=9) and a prospective cohort study (n=1). There were also three qualitative studies and one randomized controlled trial. Eight systematic reviews were included in this evidence brief to provide a global comparison (Table S2).

### Intention

Intention to accept COVID-19 boosters has decreased. Between January and October 2023, 38%–67% of individuals surveyed intended to receive a booster ((4–6)), which is lower than the intention from August 2021 to December 2022, when 61%–89% intended to receive a booster ((7–18)). Two of these studies from October 2021 to July 2022 suggested that 62%–64% of respondents were willing to receive a COVID-19 booster annually ((7,17)). The most recent study, conducted in October 2023, suggests that intention to get a booster in fall 2023 had decreased substantially since 2021 and was highest in British Columbia (45%) and lowest in Ontario (35%), Saskatchewan/Manitoba (35%) and Atlantic Canada (33%) ((5)). Across studies, individuals with more doses of COVID-19 vaccines were more likely to accept additional doses ((13,17,19)). In comparison, booster intention ranged from 56%–98% in studies captured by the global systematic reviews, which included literature published between November 2020 and February 2023 ((20–23)).

Intention of parents/guardians to vaccinate their children varied across four studies. A survey from Manitoba conducted between August and September 2022 reported that 44% of parents/guardians were likely to have their 12–17-year-old child receive a booster vaccine ((18)). A Canada-wide survey conducted from November to December 2022 reported that 30% of parents with children aged 12–17 years indicated that their children had received three doses of a COVID-19 vaccine. Among parents with children in this age group that had received two doses, 21% intended to have their child receive a third dose and 24% were unsure ((19)). The same survey reported that 17% of parents with children 5–11 years old indicated that their children had received three doses, and among parents with children in this age group that had received two doses, 52% intended to have their child receive a third dose and 17% were unsure ((19)). Intentions to receive a booster were higher during the rollout of the primary series of COVID-19 vaccines to children in a Canada-wide survey; from November 2021 to February 2022, 80.6% of parents/guardians intended for their children aged 12–17 years to receive a booster ((12)). At the beginning of the COVID-19 vaccine rollout to children, from October to November 2021, parents willing or undecided about vaccinating their children with the primary series reported general acceptance of booster doses (57.8%) and annual COVID-19 vaccination (56.4%) ((24)). None of the global systematic reviews included intentions of parents/guardians to get a booster dose for their children for comparison.

Intention to receive a COVID-19 booster was different across population subgroups, including those that have allergies, use illicit drugs, Indigenous people, immigrants, visible minorities and between sexes. A survey conducted between October 2022 and January 2023 among individuals with allergies 6–18 months post initial COVID-19 vaccination, found that 52%–57% would get a booster dose if the government or a doctor recommended it ((25)). Among a sample of vaccinated people who use illicit drugs in Canada, intention to receive a booster was 42% between March and October 2022 ((26)). Two Canada-wide studies (July–December 2022) reported that Indigenous people were slightly less likely to intend to receive additional doses compared to non-Indigenous people (38%–82% vs. 49%–89%, respectively) ((12,13)). Intention among immigrants and non-immigrants to receive a booster was similar (89.9% vs. 88.9%) between November 2021 and February 2022 ((12)). The same survey also found that visible minorities that identified as Black (76.9%) and Latin American (78.6%) were less likely to accept a booster and those that identified as Asian (91.3%–100%) were more likely compared to non-visible minorities (89%) (12)). In the same survey, LGBTQ2+ respondents were more likely than non-LGBTQ2+ respondents to intend to receive a booster (93.9% vs. 88.8%) (12)). Conflicting results were reported on whether women were more likely to accept a booster compared to men; women had higher intention in one study conducted between November 2021 and February 2022 (12)) and men had higher intentions in two studies conducted between September 2021 and March 2023 ((4,14)). In comparing these outcomes with global systematic reviews, conflicting results on whether men or women were more likely to accept a booster were also reported (22)). No other similar outcomes for comparison on intention to receive a booster dose were identified.

### Barriers and facilitators

**Figure 1:**
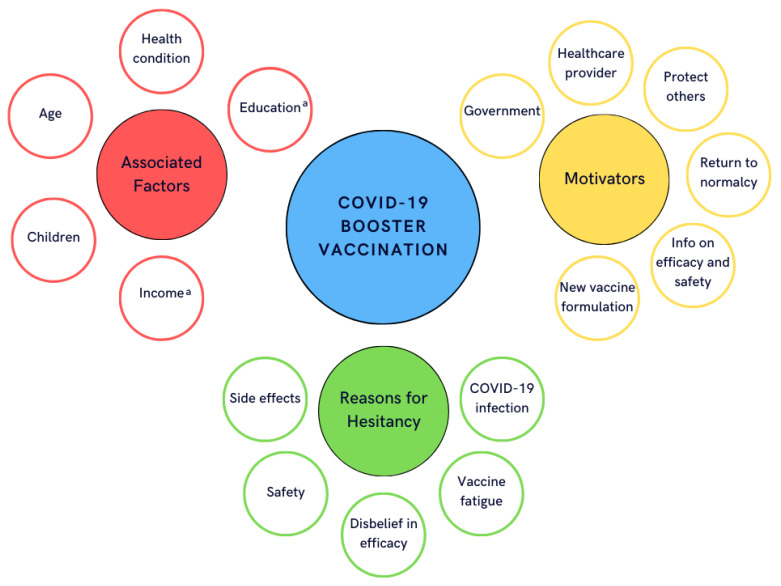
Bubble diagram of the most common barriers and facilitators of getting a COVID-19 booster vaccination, including associated factors^a^, motivators and reasons for hesitancy reported in the 21 Canadian primary literature studies ^a^ Income and education were reported to be factors associated with intention to vaccinate in studies prior to March 2023, but the most recent survey (October 2023) found no association

Barriers and facilitators regarding intention and uptake to receive boosters (**Figure 1**) were similar to accepting first and second doses of the vaccine ((27)). Factors positively associated with intention to receive boosters and uptake of boosters were older age ((4–7,12–14,17,28)), chronic health conditions ((7,12,28)), not having children ((28,29)), belief in vaccine efficacy ((29)), agreement with government decision-making ((29)), no history of a previous COVID-19 infection ((28)), being a past voter for the Liberal/Democrat parties ((16)), living in a larger/populated area ((4)) and having less vaccine fatigue ((6)). Studies between October 2021 and March 2023 reported that higher education ((4,7,12,29)) and higher income ((8,13,29)) were positively associated with higher intention and uptake to receive a booster. However, the most recent survey in October 2023 suggested that intention to get a COVID-19 booster was no longer associated with education and income groups ((5)).

Other motivators for booster intention and uptake were government recommendations ((7,28)); healthcare provider recommendations ((7,28)); personal and/or family health reasons ((7)); helping to protect others around them ((13,19,26,28,30)); emergence of new, more severe, variants ((19)); likelihood of exposure to COVID-19 ((18)); a return to normalcy ((13,28)); having information about efficacy and safety of the vaccine ((18,28)); and having new variant-specific vaccine formulations ((13,19)). Social media was identified as a decision influencer in three studies ((7,26,30)).

The main reasons for being unlikely to accept a COVID-19 booster vaccine included concerns about short and long-term side-effects ((5,6,13,19,25,28,30)), concern about the safety of receiving multiple/mixed brand doses ((4,26)), belief that a booster dose would not offer extra protection/help curb the spread ((4,6,13,19,26,31)), belief that too many doses were required, or vaccine fatigue ((4,6,13)), and belief they did not need the booster if they already had COVID-19 ((4,13)). One study (July 2022) reported that those concerned with the long-term effects of boosters were more likely to be female, less than 55 years old and not fully vaccinated or vaccinated but not boosted ((11)). Recommendations suggested for making booster vaccinations easier to obtain included walk-in appointments, provision of childcare or family appointments and paid time off from work ((7)).

Findings from the global systematic reviews were similar to that of the Canadian studies. Factors positively associated with booster intention and uptake included older age, male gender, higher education, higher income, being married, White/Asian/Hispanic ethnicity, geography (country, region and residency), history of other vaccinations and history of chronic disease ((20–22,32,33)). Previous COVID-19 infection was negatively associated with intention to have the booster dose ((21,22)), but one review found it to be positively associated with actual uptake ((21)). Motivators for booster intention and uptake were trust in vaccine effectiveness, perceived susceptibility, perceived severity and trust in authorities ((21,22,32–34)). Reasons for hesitancy included concern about adverse reactions, concerns about safety and efficacy and skepticism/distrust/conspiracy theories ((20,22,33)). Literature up to November 2022 suggested that a combined influenza and COVID-19 booster vaccine may improve the uptake of boosters ((35)).

### Attitudes and knowledge

In early 2022 (January to April), 60%–81% of Canadians believed that getting booster doses when necessary was effective at providing protection from the virus, protecting against serious illness or death or slowing the spread of virus ((11,17,36,37)). While both unvaccinated and third dose recipients in January 2022 believed they will be exposed to and infected by Omicron no matter what they do (53% vs. 54%), third dose recipients were more likely than unvaccinated to believe that if they caught COVID-19 it could be severe and/or deadly (17% vs. 7%) ((38,39)). In March 2022, a greater proportion of booster dose recipients rated their COVID-19 vaccine knowledge as very good (23%) compared to respondents who had not received a booster dose (14%) (*p*≤0.01) ((29)).

Booster dose recipients between January to March 2022 had higher trust in federal and provincial government decision-making regarding COVID-19 vaccines ((29)) and COVID-19 restrictions ((38)). However, between February and August 2022, even among those that were boosted, there was some skepticism of pharmaceutical companies, government and public health decisions and policies ((30,40)).

A randomized controlled trial looking at strategies to get people booster doses, conducted between January and February 2022, reported that participants would be less likely to get the booster if they were automatically enrolled for an appointment compared to a control condition where they initiate their own booster appointment ((41)). There was high agreement (75%) for the co-administration of COVID-19 and influenza or other routine vaccines among survey participants who were willing to receive a booster in October to November 2021 ((7)). None of the global systematic reviews included similar outcomes for comparison.

## Discussion

This evidence brief provides insight into the facilitators, barriers and hesitancy to accepting COVID-19 booster doses among Canadians between 2021 and 2023. There were no major differences observed when contrasted with the global systematic reviews. The included Canadian studies consistently reported a reduction in the intention and uptake of COVID-19 boosters between 2021, when booster doses were first recommended, and 2023. The studies captured suggest attributes of the population who are willing to accept boosters but do not give us insight into the attributes of the population whose intentions have changed as pandemic response activities have been scaled back or stopped over the last two years. These insights were also not found in any of the included global evidence syntheses.

Both the Canadian literature and global systematic reviews consistently reported that older age is positively associated with intention/uptake of a COVID-19 booster, and individuals are motivated by government/healthcare provider recommendations and the notion that they are helping to protect others ((20,22,33,42)). Between 2021 and 2023, federal/provincial/territorial public health response activities have scaled back in Canada and there has been a reduction in the general public’s focus on COVID-19. As a result, there has likely been a decrease over time in the positive impact that messaging from trusted sources had on the intentions and behaviours of individuals towards COVID-19 boosters ((43)). In addition, recommendations for boosters have varied in time and between provinces, which may have had an impact on intention/uptake of the vaccine ((2,44)). In Canada, hesitancy due to concerns regarding side effects of the vaccine and doubt in the vaccine’s efficacy continues to be a challenge and likely did not improve given the reduced public health messaging noted above. Taking these observations into account, as well as the differences in intention noted among various subgroups in Canada, will hopefully guide more tailored strategies and future public health vaccination campaigns to encourage COVID-19 booster vaccination among the Canadian population.

The evidence summarized in this evidence brief is considered to be at high to moderate risk of bias depending on the sample size and whether the sample represents the target population, as well as how well the survey tool can measure the outcome(s) of interest (e.g., whether it was informed by formative research, validated and pretested prior to implementation). Although a formal risk of bias evaluation was not conducted, the representativeness of the sampling frame, low response rates and issues with social desirability bias influencing key results were common across the observational studies. There was limited evidence on intentions and uptakes in underrepresented populations, including visible minorities, Indigenous people, children, LGBTQ2+ individuals and across genders and varying socioeconomic status. Most studies used online or telephone surveys, which may limit participation from segments of the population due to lack of access. Thus, the extent to which the findings can be applied to the target population should be considered. While many studies in this evidence brief show similar trends, the conclusions could change over time and with additional research, larger sample sizes and different sampling strategies and data collection tools.

Key topic areas for future research are intentions and reasons for hesitancy and refusal in high-risk and underserved populations, comparisons between countries and studies that identify effective interventions that would encourage individuals to stay up-to-date on the National Advisory Committee on Immunization’s COVID-19 vaccine recommendations ((2)). As the virus continues to circulate and public health responses have been scaled back to a normal level of service, understanding intentions to get vaccinated and hesitancies for accepting a booster dose remains crucial to improving booster uptake in the face of waning immunity, more transmissible variants and other public health emergencies requiring vaccination strategies.

## Conclusion

It is likely that the reduction in COVID-19 booster intentions in 2023 is related to many factors, including pandemic fatigue and the desire to move past the events of the pandemic. There is now less pressure on the community, due to reduced messaging and media coverage, to be aware of COVID-19 and to get boosters when they are recommended, as public health response activities at all levels of government have been scaled back to normal or almost normal operation. Poor vaccine uptake is not a new issue in public health; however, it would be prudent to focus on improving interventions and communication strategies to provide tailored messaging about what, when and why vaccines are needed to encourage vaccination in the general population and in underserved communities. The result of this evidence brief can inform the development of new public health strategies and prioritization of new research to address the existing knowledge gaps.

## Appendix Data Availability

All relevant data are included in the paper or its Supplementary Information (Supplementary Files 1–3): https://doi.org/10.17605/OSF.IO/8YH7R

## References

[1] Public Health Agency of Canada. Canadian COVID-19 vaccination coverage report. Ottawa, ON: PHAC; 2024. [Accessed 2024 Mar 28]. https://health-infobase.canada.ca/covid-19/vaccination-coverage/

[2] Government of Canada. An Advisory Committee Statement (ACS) National Advisory Committee on Immunization (NACI). Guidance on the use of COVID-19 vaccines during the fall of 2024. Ottawa, ON: Government of Canada; 2024. https://www.canada.ca/en/public-health/services/publications/vaccines-immunization/national-advisory-committee-immunization-guidance-covid-19-vaccines-fall-2024.html

[3] Corrin T, Ayache D, Baumeister A, Young K, Pussegoda K, Ahmad R, Waddell L. COVID-19 literature surveillance-A framework to manage the literature and support evidence-based decision-making on a rapidly evolving public health topic. Can Commun Dis Rep 2023;49(1):5–9. 10.14745/ccdr.v49i01a02 36815866 PMC9902036

[4] Institut national de santé publique du Québec. Pandémie et vaccination - Résultats du 4 avril 2023. Québec, QC: INSPQ; 2023. https://www.inspq.qc.ca/covid-19/sondages-attitudes-comportements-quebecois/vaccination-4-avril-23

[5] IPSOS. Four in Ten (40%) Canadians Do Not Intend to Get a COVID-19 Booster Vaccine Nor a Flu Shot. Vancouver, BC: IPSOS; 2023. https://www.ipsos.com/en-ca/four-in-ten-canadians-do-not-intend-get-covid-19-booster-vaccine-nor-flu-shot

[6] Canadian Pharmacists Association. Vaccine intentions among Canadians August 2023 - OMNI Survey Results. 2023. https://www.pharmacists.ca/cpha-ca/assets/File/cpha-on-the-issues/CPhA-Cold-and-flu-season-survey-Aug2023-Release-deck.pdf

[7] Reifferscheid L, Lee JS, MacDonald NE, Sadarangani M, Assi A, Lemaire-Paquette S, MacDonald SE. Transition to endemic: acceptance of additional COVID-19 vaccine doses among Canadian adults in a national cross-sectional survey. BMC Public Health 2022;22(1):1745. 10.1186/s12889-022-14025-8 36104675 PMC9473459

[8] Lazarus JV, Wyka K, White TM, Picchio CA, Gostin LO, Larson HJ, Rabin K, Ratzan SC, Kamarulzaman A, El-Mohandes A. A survey of COVID-19 vaccine acceptance across 23 countries in 2022. Nat Med 2023;29(2):366–75. 10.1038/s41591-022-02185-4 36624316

[9] IPSOS. Global Attitudes on COVID-19 Vaccine Booster Shots. Vancouver, BC: IPSOS; 2021. https://www.ipsos.com/sites/default/files/ct/news/documents/2021-09/Global-attitudes-about-COVID-19-Vaccine-Booster-Shots-Sept%202021.pdf

[10] IPSOS. Two in Three (67%) Canadians Believe that a Fully Vaccinated Population Won’t be Enough to Stop the Spread of Omicron. Vancouver, BC: IPSOS; 2022. https://www.ipsos.com/en-ca/news-polls/Two-Three-Canadians-Believe-Fully-Vaccinated-Population-Not-Enough-Stop-Omicron

[11] IPSOS. Continued Strong Support for COVID-19 Boosters Among Canadians. Vancouver, BC: IPSOS; 2022. https://www.ipsos.com/en-ca/news-polls/continued-strong-support-for-boosters-among-canadians

[12] Statistics Canada. Archived – Canadians’ health and COVID-19, by region, age, gender and other characteristics, inactive. Ottawa, ON: StatCan; 2022. [Accessed 2024 Mar 29]. https://www150.statcan.gc.ca/t1/tbl1/en/cv.action?pid=1310080901

[13] Public Health Agency of Canada. The Impact of the Pandemic Experience on Future Vaccine-Related Intentions and Behaviours (2022). Ottawa, ON: PHAC; 2023. https://publications.gc.ca/collections/collection_2023/aspc-phac/H14-432-2023-eng.pdf

[14] Nanos Research. A strong majority of Canadians say they will definitely take the COVID-19 vaccine booster shot when available. 2021. https://nanos.co/wp-content/uploads/2022/01/2021-2045-Globe-December-Populated-Report-Booster-with-Tabs.pdf

[15] Nanos Research. Strong majority of Canadians show interest in getting a COVID-19 vaccination booster shot. 2021. https://nanos.co/wp-content/uploads/2021/10/2021-1981-CTV-September-Populated-report-Powerplay-with-tabs.pdf

[16] Angus Reid Institute. Kids and COVID: Half of Canadian parents with children aged 5-11 ready to vaccinate their little ones ASAP. 2021. https://angusreid.org/wp-content/uploads/2021/10/2021.10.13_COVID_October_.pdf

[17] Angus Reid Institute. COVID-19: Half want boosters ASAP, but two-in-five among vaccinated say they’re not sold on another shot. 2022. https://angusreid.org/covid-19-canada-booster-vaccine-skepticism/

[18] Manitoba Health. COVID-19 Vaccine Planning for Fall. Winnipeg, MB: Manitoba Health; 2022. https://manitoba.ca/asset_library/en/proactive/20222023/covid-19-for-fall-en.pdf

[19] Impact Canada. COVID-19 Snapshot Monitoring. 2022. [Accessed 2024 Mar 28]. https://impact.canada.ca/en/cosmo-canada

[20] Galanis P, Vraka I, Katsiroumpa A, Siskou O, Konstantakopoulou O, Katsoulas T, Mariolis-Sapsakos T, Kaitelidou D. First COVID-19 booster dose in the general population: A systematic review and meta-analysis of willingness and its predictors. Vaccines (Basel) 2022;10(7):1097. 10.3390/vaccines10071097 35891260 PMC9323526

[21] Abdelmoneim SA, Sallam M, Hafez DM, Elrewany E, Mousli HM, Hammad EM, Elkhadry SW, Adam MF, Ghobashy AA, Naguib M, Nour El-Deen AE, Aji N, Ghazy RM. COVID-19 vaccine booster dose acceptance: systematic review and meta-analysis. Trop Med Infect Dis 2022;7(10):298. 10.3390/tropicalmed7100298 36288039 PMC9611447

[22] Limbu YB, Huhmann BA. Why some people are hesitant to receive COVID-19 boosters: A systematic review. Trop Med Infect Dis 2023;8(3):159. 10.3390/tropicalmed8030159 36977160 PMC10054177

[23] McKinley CJ, Limbu Y. Promoter or barrier? Assessing how social media predicts Covid-19 vaccine acceptance and hesitancy: A systematic review of primary series and booster vaccine investigations. Soc Sci Med 2024;340:116378. 10.1016/j.socscimed.2023.116378 38042027

[24] Humble RM, Sell H, Wilson S, Sadarangani M, Bettinger JA, Meyer SB, Dubé È, Lemaire-Paquette S, Gagneur A, MacDonald SE. Parents’ perceptions on COVID-19 vaccination as the new routine for their children ≤ 11 years old. Prev Med 2022;161:107125. 10.1016/j.ypmed.2022.107125 35792197 PMC9250244

[25] Stehlin F, Khoudja RY, Al-Otaibi I, ALMuhizi F, Fein M, Gilbert L, Tsoukas C, Ben-Shoshan M, Copaescu AM, Isabwe GA. COVID-19 booster vaccine acceptance following allergy evaluation in individuals with allergies. J Allergy Clin Immunol Pract 2024;12(1):242–245.e2. 10.1016/j.jaip.2023.09.037 37802251

[26] Ali F, Kaura A, Russell C, Bonn M, Bruneau J, Dasgupta N, Imtiaz S, Martel-Laferrière V, Rehm J, Shahin R, Elton-Marshall T. Identifying barriers and facilitators to COVID-19 vaccination uptake among People Who Use Drugs in Canada: a National Qualitative Study. Harm Reduct J 2023;20(1):99. 10.1186/s12954-023-00826-6 37516836 PMC10387201

[27] Public Health Agency of Canada. Evergreen Rapid Review on COVID-19 Vaccine Attitudes and Uptake in Canada - Update 11. Ottawa, ON: PHAC; 2021. https://www.canada.ca/en/public-health/services/diseases/2019-novel-coronavirus-infection/canadas-reponse/summaries-recent-evidence/evergreen-rapid-review-vaccine-attitudes-uptake-update-11.html

[28] Léger C, Deslauriers F, Gosselin Boucher V, Phillips M, Bacon SL, Lavoie KL. Prevalence and motivators of getting a COVID-19 booster vaccine in Canada: results from the iCARE study. Vaccines (Basel) 2023;11(2):291. 10.3390/vaccines11020291 36851169 PMC9960725

[29] Leigh JP, FitzGerald EA, Moss SJ, Brundin-Mather R, Dodds A, Stelfox HT, Dubé È, Fiest KM, Halperin D, Ahmed SB, MacDonald SE, Straus SE, Manca T, Kamstra JN, Soo A, Longmore S, Kupsch S, Sept B, Halperin S. Factors affecting hesitancy toward COVID-19 vaccine booster doses in Canada: a cross-national survey. Can J Public Health 2024;115(1):26–39. 10.17269/s41997-023-00823-z 37991692 PMC10853155

[30] Zhu P, Tatar O, Haward B, Steck V, Griffin-Mathieu G, Perez S, Dubé È, Zimet G, Rosberger Z. Examining an altruism-eliciting video intervention to increase COVID-19 vaccine intentions in younger adults: A qualitative assessment using the realistic evaluation framework. Vaccines (Basel) 2023;11(3):628. 10.3390/vaccines11030628 36992212 PMC10056235

[31] IPSOS. BC Booster Shots: Fewer than Half (44%) of British Columbians with Two Doses of a COVID-19 Vaccine Plan to Get Their Booster Shot as Soon as Available. Vancouver, BC: IPSOS; 2022. https://www.ipsos.com/sites/default/files/ct/news/documents/2022-02/BCPhA_Boosters-Factum-2022-02-09-v1_2.pdf

[32] Ayyalasomayajula S, Dhawan A, Karattuthodi MS, Thorakkattil SA, Abdulsalim S, Elnaem MH, Sridhar S, Unnikrishnan MK. A systematic review on sociodemographic, financial and psychological factors associated with COVID-19 vaccine booster hesitancy among adult population. Vaccines (Basel) 2023;11(3):623. 10.3390/vaccines11030623 36992207 PMC10051942

[33] Yazdani Y, Pai P, Sayfi S, Mohammadi A, Perdes S, Spitzer D, Fabreau GE, Pottie K. Predictors of COVID-19 vaccine acceptability among refugees and other migrant populations: A systematic scoping review. medRxiv 2023. [Accessed 2024 Mar 28]. 10.1101/2023.09.15.23295608 10.1101/2023.09.15.23295608 PMC11226018 38968187

[34] Limbu YB, Gautam RK. How well the constructs of health belief model predict vaccination intention: A systematic review on COVID-19 primary series and booster vaccines. Vaccines (Basel) 2023;11(4):816. 10.3390/vaccines11040816 37112728 PMC10141697

[35] Tzenios N, Tazanios ME, Chahine M. Combining influenza and COVID-19 booster vaccination strategy to improve vaccination uptake necessary for managing the health pandemic: A systematic review and meta-analysis. Vaccines (Basel) 2022;11(1):16. 10.3390/vaccines11010016 36679863 PMC9860577

[36] Leger. North American Tracker - January 10th, 2022. 2022. https://leger360.com/wp-content/uploads/2024/02/Legers-North-American-Tracker-January-10th-2022_V2.pdf

[37] Government of Canada. Childhood COVID-19 Immunization Coverage Survey (CCICS): 2022 results. Ottawa, ON: Government of Canada; 2023. https://www.canada.ca/en/public-health/services/immunization-vaccines/vaccination-coverage/childhood-covid-19-immunization-coverage-survey-2022-results.html

[38] Angus Reid Institute. Omicron Inevitability? 55% say they’ll be infected regardless of precautions; two-in-five would end all restrictions. 2022. https://angusreid.org/wp-content/uploads/2022/01/2022.01.13_COVID_inevitability.pdf

[39] Angus Reid Institute. Unconcerned about Omicron: More than four-in-five now believe a COVID-19 infection would be mild, manageable. 2022. https://angusreid.org/wp-content/uploads/2022/01/2022.01.26_COVID_Unconcerned_about_Omicron.pdf

[40] Thaivalappil A, Young I, MacKay M, Pearl DL, Papadopoulos A. A qualitative study exploring healthcare providers’ and trainees’ barriers to COVID-19 and influenza vaccine uptake. Health Psychol Behav Med 2022;10(1):695–712. 10.1080/21642850.2022.2106231 35957955 PMC9359157

[41] Banerjee S, Hunter A, John P, Koenig R, Lee-Whiting B, Loewen P, McAndrews J, Nyhan B, Savani M. Thinking about default enrollment lowers vaccination intentions and public support in G7 countries. PNAS Nexus 2024;3(4):093. https://academic.oup.com/pnasnexus/article/3/4/pgae093/7614389 10.1093/pnasnexus/pgae093 PMC10997051 38585340

[42] Government of Canada. Evidence brief on attitudes and acceptance of COVID-19 booster doses. Ottawa, ON: Government of Canada; 2022. https://www.canada.ca/en/public-health/services/diseases/2019-novel-coronavirus-infection/canadas-reponse/summaries-recent-evidence/evidence-brief-attitudes-acceptance-covid-19-booster-doses.html

[43] Canadian Institute for Health Information. Canadian Data Set of COVID-19 Interventions - Data Tables. Ottawa, ON: CIHI; 2022. [Accessed 2024 Aug 13]. https://www.cihi.ca/en/canadian-covid-19-intervention-timeline

[44] Institut national de santé publique du Québec. Administration de doses de rappel du vaccin contre la COVID-19: recommandations pour l’automne 2023. Québec, QC: INSPQ; 2023. https://www.inspq.qc.ca/publications/3367

